# Single-cell analysis of age-related changes in leukocytes of diabetic mouse hindpaws

**DOI:** 10.1007/s00018-024-05128-z

**Published:** 2024-03-19

**Authors:** James M. Nichols, Hoang Vu Pham, Eric F. Lee, Rajasekaran Mahalingam, Andrew J. Shepherd

**Affiliations:** The MD Anderson Pain Research Consortium and the Laboratories of Neuroimmunology, Department of Symptom Research, Division of Internal Medicine, The University of Texas MD Anderson Cancer Center, Unit 1055, 6565 MD Anderson Boulevard, Houston, TX 77030 USA

**Keywords:** Type 2 diabetes, Skin, Macrophage, Single-cell RNA sequencing

## Abstract

Complications associated with Type 1 and Type 2 diabetes, such as diabetic peripheral neuropathy and diabetic foot ulcers, are a growing health-care concern. In addition, this concern increases as diabetic patients age due to their increased susceptibility to complications. To address this growing problem, it is important to understand fluctuations in physiology which lead to pathological changes associated with the metabolic disturbances of diabetes. Our study explores dysregulation of immune cell populations in the hindpaws of healthy and diabetic mice at 12 and 21 weeks of age using single-cell RNA sequencing to provide insight into immune disruptions occurring in the distal limb during chronic diabetes. In 21-week-old *Lepr*^*db/db*^ mice, increases were seen in mast cells/basophils, dermal γδ T cells, heterogeneous T cells, and Type 2 innate lymphoid cells. In addition, macrophages represented the largest cluster of immune cells and showed the greatest increase in genes associated with immune-specific pathways. Sub-clustering of macrophages revealed a bias toward angiogenic Lyve1^+^MHCII^lo^ macrophages in the hindpaws of 21-week-old diabetic mice, which corresponded to an increase in Lyve1^+^ macrophages in the hindpaws of 21-week-old diabetic mice on histology. Our results show that in Type 2 diabetes, the immunological function and phenotype of multiple immune cell types shift not only with metabolic disturbance, but also with duration of disease, which may explain the increased susceptibility to pathologies of the distal limb in patients with more chronic diabetes.

**Supplementary Information:**

The online version contains supplementary material available at 10.1007/s00018-024-05128-z.

## Introduction

The incidence and prevalence of diabetes has increased in the last several decades, with estimates putting the number of people with diabetes at almost half a billion worldwide [1]. This rise has also increased the incidence of two common complications of diabetes: diabetic peripheral neuropathy (DPN) and diabetic foot ulcers (DFUs) [1, 2]. DPN often presents as a distal symmetric polyneuropathy which affects approximately half of all people with diabetes and starts as a tingling, burning, or numb sensation in the fingertips and toes that progresses proximally over time [2, 3]. DPN is one of many types of neuropathy affecting diabetic patients, which can range in symptoms from autonomic dysregulation to oculomotor dysfunction, and these neuropathies are thought to be driven primarily by high blood glucose levels, vascular disruption, and impaired nerve function [3–6]. On the contrary, DFUs are the result of impaired wound healing in diabetic patients and lead to increased rates of infection, hospitalization, lower limb amputation, and death [7]. DFU represents one of the leading causes of non-traumatic lower limb amputations and is often preceded by DPN, as the lack of sensation in the distal limb increases the risk for injury [1, 8]. Since these conditions have been shown to have immunological dysregulation associated with their pathology, and inflammaging has been shown to contribute to a plethora of diseases including diabetes [8–18], here we examine the immune system in the distal hind limb of healthy and diabetic mice at variable ages to gain a holistic view of the immunological drivers behind these complications.

The metabolic dysregulation associated with diabetes alters many of the roles carried out by immune cells. In macrophages, many functions have been shown to be altered under diabetic conditions including phagocytosis [19–21], wound healing [16], and cytokine expression [22–24]. This suggests that there are pleiotropic effects of diabetes on the secretory output and function of macrophages. Alongside macrophages, dysregulation of T cells also occurs. Cutaneous T cells expressing αβ and γδ T cell receptors play vital roles in the dermal and epidermal microenvironment, and impairment of the normal physiological functions of these cells contributes to compromised wound healing [25]. Furthermore, T-bet-expressing B cells have been shown to play a role in the inflammatory response of rodents in the high-fat diet-induced model of Type 2 diabetes mellitus (T2DM) as well as in diabetic patients, a response supported by invariant natural killer T cells [26].

Based on the understanding that metabolic disturbances in diabetes have wide-ranging effects on leukocyte populations, phenotype, and function, we utilized single-cell RNA sequencing to survey the immune cells present within the hind foot of diabetic and healthy mice. Importantly, our study also incorporates 12-week-old and 21-week-old mice without injury to the distal limb to more accurately capture dysregulation of the immune system driven primarily by T2DM as the disease progresses over time. From this analysis, we identify changes in macrophage populations suggestive of an increase in angiogenic M2-like macrophages in the hindpaw of diabetic mice, which we were able to confirm histologically. In addition, large shifts were noted in other cell populations from our sequencing data, with notable changes occurring in a variety of T cell populations. The data presented here advances our understanding of how the immune system of diabetic patients might change with chronicity of disease.

## Materials and methods

### Mice

Experiments were approved by the MD Anderson Institutional Animal Care and Use Committee. Male *Lepr*^*db/db*^ (DB) (C57BLKS/J-*Dock7*^*m*^ + / + *Lepr*^*db*^, Strain #: 000642) and *Lepr*^*WT/WT*^(WT) littermates were used at 12 weeks old or 21 weeks old. The mice were housed two to five per cage in a temperature (22 °C ± 1 °C)—and humidity (40–60%)—controlled room with a 12-h light/dark cycle and ad libitum access to food (Purina PicoLab^®^ Rodent Diet 5053; 20% protein, 4.5% fat) and water.

### Von Frey testing

To validate the mechanical hyposensitivity previously noted in this model [27], we performed weekly Von Frey measurements from 11 weeks of age to 21 weeks of age. Von Frey filaments (0.04–2.00 g) were presented five times to both hindpaws of WT and Lepr^db/db^ mice (*n* = 5) progressing from the smallest to the largest filament, as described previously [28, 29]. The number of responses to each filament was then used to generate an area under the curve (AUC) value for each limb, and the average of the two responses was taken.

### Blood glucose measurement

The early-onset hyperglycemia typical of this model was also confirmed using a Precision Xtra glucometer (Abbott) to measure fasting blood glucose from WT and Lepr^db/db^ mice at 12 weeks of age (*n* = 5). For blood glucose measurement, the mice were fasted for 4 h prior to being placed in a restraint, and blood was obtained from the lateral tail vein without sedation.

### Hindpaw digestion

Mice were euthanized with CO_2_ and transcardially perfused with 20 ml of cold PBS (without Ca^2+^, Mg^2+^). Both hindpaws were excised at the ankle. A scalpel was used to make four linear, full-thickness cuts between the metatarsals to facilitate digestion. The paws were digested in 5 mg/ml collagenase in RPMI/1% Pen/Strep/20 mM HEPES (Corning) for 1.5–2 h at 37 °C with agitation for every 15 min. The cell suspension and remaining tissue were filtered through a 70-µm nylon filter using a 5-ml syringe plunger to further dissociate cells from the tissues. The filter was rinsed with 2 ml of RPMI/1% Pen/Strep/20 mM HEPES and the suspension was transferred into a 15-ml tube. Ten milliliters of RPMI/1% Pen/Strep/20 mM HEPES was added to each tube to dilute the collagenase and preserve cell viability. The samples then underwent centrifugation for 5 min at 500x*g*, and the cells were resuspended in 1 ml of FACS buffer (2% heat-inactivated FBS + 0.5 mM EDTA in PBS) for cell sorting.

### Fluorescent staining and cell sorting

To block Fc receptors, cells were incubated with 0.5 µl of TruStain FcX PLUS in 50 µl FACS of buffer. Thirty minutes later, anti-CD45 APC (1.25 µl; clone 30-F11, Biolegend; 5 µg/ml) and anti-CD19 BV421 (1.5 µl; clone 6D5, Biolegend; 1.5 µg/ml) were added in 50 µl of FACS buffer. Following incubation in darkness for 30 min at room temperature (RT), the cells were washed and resuspended in 300 µl of FACS buffer containing 0.3 µl of Sytox Green viability dye (Biolegend; 5 µM). A BD FACS Aria cell sorter was used to isolate CD45^+^ cells into RPMI + 2% FBS and resuspend them in 50 µl of PBS + 2% FBS. CD19^+^ signal was used to assess bone marrow contamination. In all samples, CD19^+^ cells represented less than 5% of total CD45^+^ cells, indicating minimal bone marrow contamination. Pooled CD45^+^ cell suspensions (*n* = 2–3 mice per group) were transferred to the MD Anderson Advanced Technology Genomics Core (ATGC, CA016672) for single-cell library preparation. Cell density and viability were determined using a Countess II FL auto-counter. Single cells were captured (~ 6000–9000 cells/sample) using Chromium Controller (10X Genomics), and sequencing was done by Novaseq 6000.

### ScRNA-seq quality control and data processing

Raw sequencing files were aligned utilizing CellRanger 6.0 (10 × Genomics). Each sequencing file was aligned to an indexed mouse genome (mm10), and gene count matrices were generated. Downstream analysis was performed with the R package Seurat 4.0. Across all samples, cells with < 200 genes and > 10% mitochondrial genes were removed from subsequent analysis. Ambient RNA removal was performed using the “decontx” algorithm in the celda package (v.1.1.6). The decontx algorithm applies a heuristic approach to define the clustering and then empirically estimate the distribution of ambient RNA. After filtering ambient RNA, the count matrix was used for clustering analysis using the Seurat functions NormalizeData, FindVariableFeatures (with 2,000 features), ScaleData, and RunPCA. The top 15 principal components (PCs) were considered for defining cell clusters using the FindNeighbours and FindClusters (resolution = 0.3) functions. After clustering each sample, doublets and multiplets were filtered using the DoubletFinder program with default settings.

### ScRNA-seq integration

Each sample was processed separately and then integrated to identify integrated clusters and cell types. The integration approach searches for pairs of cells in a similar biological state across the samples (anchors) through the FindIntegrationAnchors function and combines the data by using the IntegrateData function. Principal component analysis (top 20 PCs) on the integrated data with significant, highly variable genes was performed using RunPCA, followed by FindNeighbors and FindClusters (resolution = 0.4), to obtain the final 19 clusters.

### Cluster annotation

All clusters were annotated through manual analysis based on differential gene expression of each cluster. The FindAllMarker function with default parameters was used to obtain differentially expressed genes for each cluster. The top marker genes from each cluster were used to annotate cell types using a previous single-cell study of cutaneous mouse leukocytes [30] and the ImmGen database [31]. All the 19 clusters were annotated into 14 cell types. After removal of two small clusters (annotated as stromal and cell cycle-specific cells) from the dataset, re-clustering generated the final 12 immune cell types (Supplemental Fig. 1).

### Cell–cell communication analysis

Cell–cell communication analysis was conducted by assessing the expression of specific receptor–ligand pairs in various cell types derived from scRNA-seq data. The Cellchat tool, as described by Jin et al. [32], was employed in this study. The standard Cellchat workflow was followed to identify potential alterations or inductions in cell–cell communication networks within the 12WT, 12DB, 21WT, and 21DB samples. Input data for the analysis comprised normalized counts from all samples, along with cell clusters obtained from the Seurat program. Default parameters were used for preprocessing functions such as identifyOverExpressedGenes, identifyOverExpressedInteractions, and projectData. The Secreted Signalling pathways database, along with precompiled mouse protein–protein interactions, served as a priori network information. Subsequently, functions including computeCommunProb, computeCommunProbPathway, and aggregateNet were applied to calculate the cell–cell communication.

### Lyve1 clustering analysis

All macrophages were designated Lyve1 positive or negative. Differentially expressed genes were calculated between these two groups using the FindMarkers function. The dataset then underwent supervised cluster analysis, resulting in the division of cells into four sub-groups based on the log-normalized expression values of Lyve1 and H2-Ab1 (MHCII) mRNAs. The sub-groups were defined as follows: Lyve1^+^MHCII^hi^ (Lyve1 > 0 & H2-Ab1 ≥ 2), Lyve1^+^MHCII^lo^ (Lyve1 > 0 & H2-Ab1 < 2), Lyve1^−^MHCII^hi^ (Lyve1 = 0 & H2-Ab1 ≥ 2), and Lyve1^−^MHCII^lo^ (Lyve1 = 0 and H2-Ab1 < 2).

### Pathway analysis

Pathway analysis was performed using the QIAGEN IPA (QIAGEN Inc., https://digitalinsights.qiagen.com/IPA) and ToppGene Suite. For comparative pathway analysis, the differentially expressed genes for all cell types in each sample were used. For the Lyve1/MHCII sub-clustering analysis, enrichment analysis was performed with Gene Ontology (GO) biological process terms. The background gene list used in these analyses is provided in Supplementary Table 1, as per [33, 34]. For the analysis of GO terms related to differentially expressed genes in Lyve1-positive and Lyve1-negative comparisons, we utilized the ClueGO plugin within Cytoscape program [35].

### Histology

#### Tissue processing

WT and *Lepr*^*db/db*^ mice at 12 and 21 weeks of age (*n* = 3–6) were euthanized with CO_2_ and perfused with cold PBS followed by 10% neutral buffered formalin (Thermo Scientific). Tissues were post-fixed overnight at 4 °C and decalcified using 10% (w/v) EDTA in PBS for 14d at 4 °C (EDTA was replaced after 7 days). The tissues were cryoprotected in 30% sucrose for 48 h at 4 °C and frozen in Tissue-Tek^®^ O.C.T. compound (Sakura). Thirty-micron sections from the distal end of metatarsals 2–4 were collected onto Superfrost Plus slides (Fisher Scientific) using a Leica CM3050S Cryostat (Leica Biosystems).

#### Immunofluorescence

Tissue sections were rinsed in PBS before adding blocking solution [PBS + 10% (v/v) goat serum (Sigma)/1% bovine serum albumin (Fisher Scientific)/0.3% Triton X-100 (Fisher)] for 1 h. Primary antibodies were then diluted in blocking solution and incubated overnight at 4 °C in a humidified slide staining chamber. Lyve1^+^ and MHCII^+^ were detected using rat anti-Lyve1 (Clone: ALY7;eBioscience; 1: 500) and rat anti-MHCII (Clone: M5/114.15.2; Novus Biologicals; 1: 500) primary antibodies, paired with rabbit anti-CD68 (polyclonal; Abcam; 1: 1000)/rabbit anti-Iba1 (polyclonal; Wako; 1: 1000), rabbit anti-CD68 (polyclonal; Abcam; 1: 500), rabbit anti-PGP9.5 (Clone: EPR4118; Abcam; 1: 200), rabbit anti-CD206/MRC1 (Clone E6T5J; Cell Signaling; 1: 500), or rabbit anti-CD31 (Clone: SP38; Invitrogen; 1: 50). T cells were detected with rat anti-CD3 (Clone: CD3-12; Abcam; 1: 100). Slides were then rinsed 10 × in PBS. Secondary antibodies (goat anti-rabbit Alexa Fluor-594 with goat anti-rat Alexa Fluor-488, or goat anti-rabbit Alexa Fluor-488 with goat anti-rat Alexa Fluor-594; 1: 500; Invitrogen) in PBS containing DAPI (500 ng/ml; Sigma) were placed on the slides and incubated for 3 h at RT. Sections were again rinsed 10 × in PBS and coverslipped with FluorSave mounting medium (Millipore).

### Microscopy

Images were captured using a Nikon Ti2 confocal microscope (Nikon Instruments Inc., Melville, NY) with a 40x (Numerical aperture:1.30) objective at 2 µm z-stacks. Images were taken from the ventral aspect of each hindpaw. Thresholds were applied to the Alexa Fluor-594 and Alexa Fluor-488 signals independently for percent areas, and thresholds were combined to generate the region of interest (ROI) used for the Pearson’s correlation of Alexa Fluor-594 and Alexa Fluor-488 signals. Quantification was performed using Nikon NIS-Elements Advanced Research Software (Nikon Instruments Inc.).

### Statistical analysis

Statistical analysis was performed using GraphPad Prism (version 10). Two-way ANOVA with Tukey’s multiple comparisons test was performed on histological data. Two-way ANOVA with Šidák’s multiple comparisons test was performed for Von Frey data. Mann–Whitney test was performed on blood glucose data. Statistical significance was determined in the analysis of differential gene expression by applying a threshold of an adjusted *p*-value < 0.05 and a fold change cutoff of 0.25.

## Results

### *Lepr*^*db/db*^ mice show early-onset hyposensitivity and hyperglycemia

**Fig. 1:**
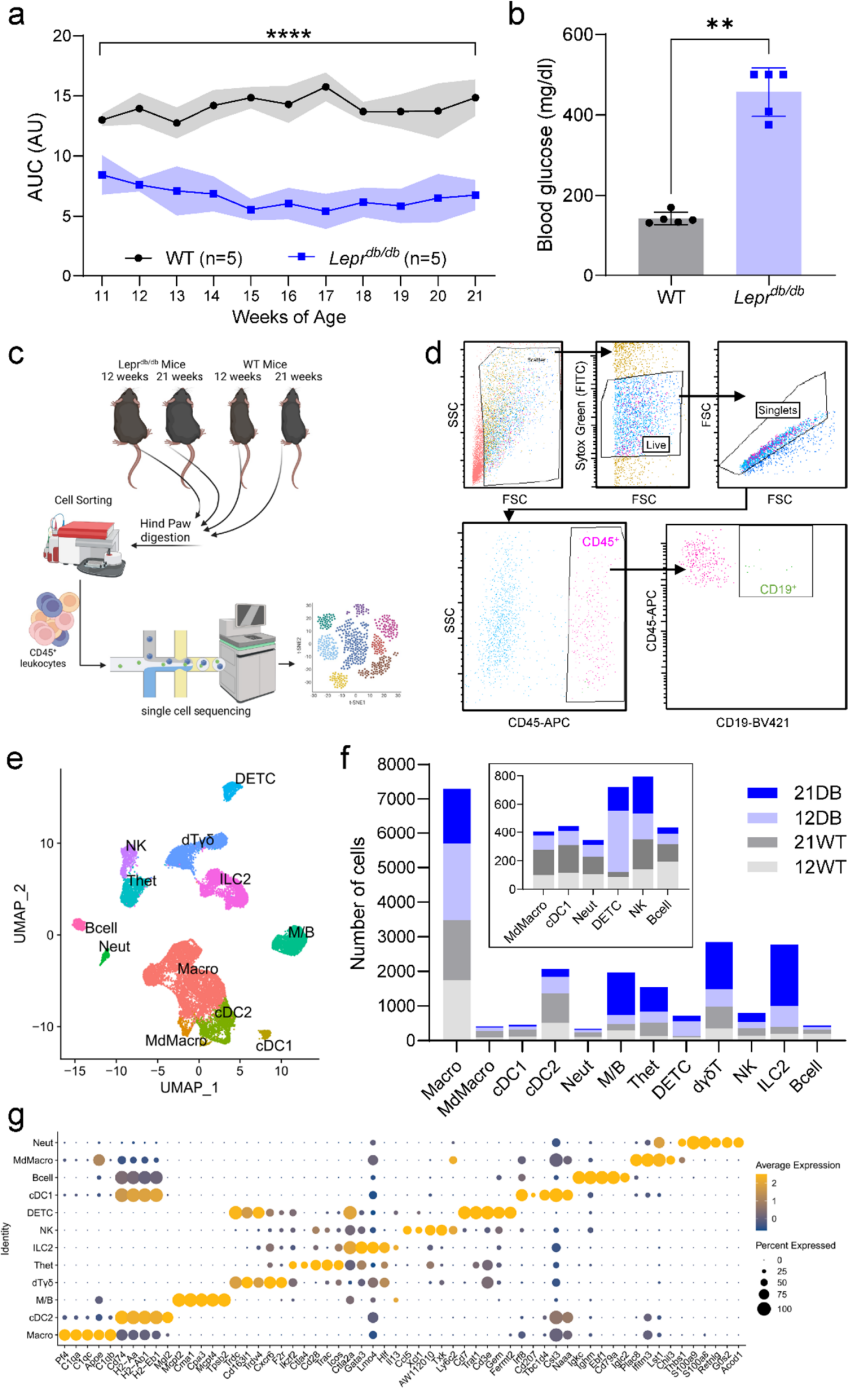
Model validation, analysis pipeline, and identification of hindpaw immune cell heterogeneity: **a** von Frey and **b** blood glucose levels were used to verify the neuropathy and metabolic phenotype of *Lepr*^*db/db*^ mice, respectively (*n* = 5). **c** Overview of the single-cell RNA-seq workflow. **d** Gating strategy for the isolation of single cells from hindpaws by flow cytometry depicting the gating system used to isolate CD45^+^ cells, which included a gate based on forward (FSC) and side (SSC) scatter, followed by a singlet gate and a CD45^+^ gate. In addition to these gates, which were used for sample collection, a CD19^+^ gate was used to check for excessive B-cell numbers as a proxy measure of bone marrow contamination. **e** Uniform Manifold Approximation and Projection (UMAP) plot of all 21,661 cells from 12-week-WT, 12-week-DB, 21-week-WT, and 21-week-DB mouse hindpaws. **f** Bar plot showing the number of cells for each cell type in each sample. Low-abundance clusters are re-plotted in the inset. **g** Dot plot showing the top 5 marker genes detected across all the 12 cell types. Data are mean ± SD; **/****p < 0.01, 0.0001, WT versus *Lepr*^*db/db*^, two-way ANOVA, Šidák / Mann–Whitney test

Von Frey testing of WT and *Lepr*^*db/db*^ mice showed significant hyposensitivity in *Lepr*^*db/db*^ mice ranging from 11 to 21 weeks of age (Fig. 1a). In addition, blood glucose of mice at 12 weeks of age confirmed a significant hyperglycemia in *Lepr*^*db/db*^ mice by 12 weeks of age (Fig. 1b).

### Single-cell analysis reveals hindpaw immune cell heterogeneity

Sequencing of CD45^+^ cells from 12- and 21-week-old WT and DB hindpaws generated an integrated dataset of 21,661 cells grouped into 12 clusters (Fig. 1c–f), defined as: macrophages (*Pf4, C1qa, C1qc, Apoe, C1qb*), type 2 conventional dendritic cells (cDC2) (*CD74, H2-Aa, H2-Ab1, H2-Eb1, Mgl1*), mast cells/basophils (M/B) (*Mcpt2, Cma1, Cpa3, Mcpt4, Tpsb2*), dermal γδ T (dT_γδ_) cells (*Trdc, Cd163l1, Trdv4, Cxcr6, F2r*), heterogeneous T (T_het_) cells (*Ikzf2, Ctla4, CD28, Trac, Icos*), Type 2 innate lymphoid cells (ILC2) (*Ctla2a, Gata3, Lmo4, Hlf, Il13*), natural killer (NK) cells (*Ccl5, xcl1, AW112010, Txk, Ly6c2*), dendritic epidermal T cells (DETC; *Cd7, Trat1, CD3e, Gem, Fermt2*), type 1 conventional dendritic cells (cDC1) cells (*Irf8, CD207, Tbc1d4, Cst3, Naaa*), B cells (*Igkc, Ighm, Ebf1, CD79a, Iglc2*), monocyte-derived macrophages (MdMacro) (*Plac8, Ifitm3, Lst1, Chil3, Thbs1*), and neutrophils (Neut) (*S100a9, S100a8, Retnlg, G0s2, Acod1*) (Fig. 1g, Supplementary Fig. 1 and Supplementary Table 2) [30]. After quality control and filtering, the number of single-cell transcriptomes was 3,946 (12-week-WT), 5,413 (12-week-DB), 4,852 (21-week-WT), and 7,450 (21-week-DB). When each sample was represented as a proportion of the integrated cluster total, variation in cell numbers in each cluster was apparent (Fig. 1e, f). Macrophages were the most abundant cell type across all samples, while mast cell/basophil (M/B), dermal γδ T cell (dT_γδ_), heterogeneous T cell (T_het_), and Type 2 innate lymphoid cell (ILC2) populations were substantially increased in 21-week-DB mice (Fig. 1f and Supplementary Table 3).

### Gene expression changes in WT and DB mice

**Fig. 2:**
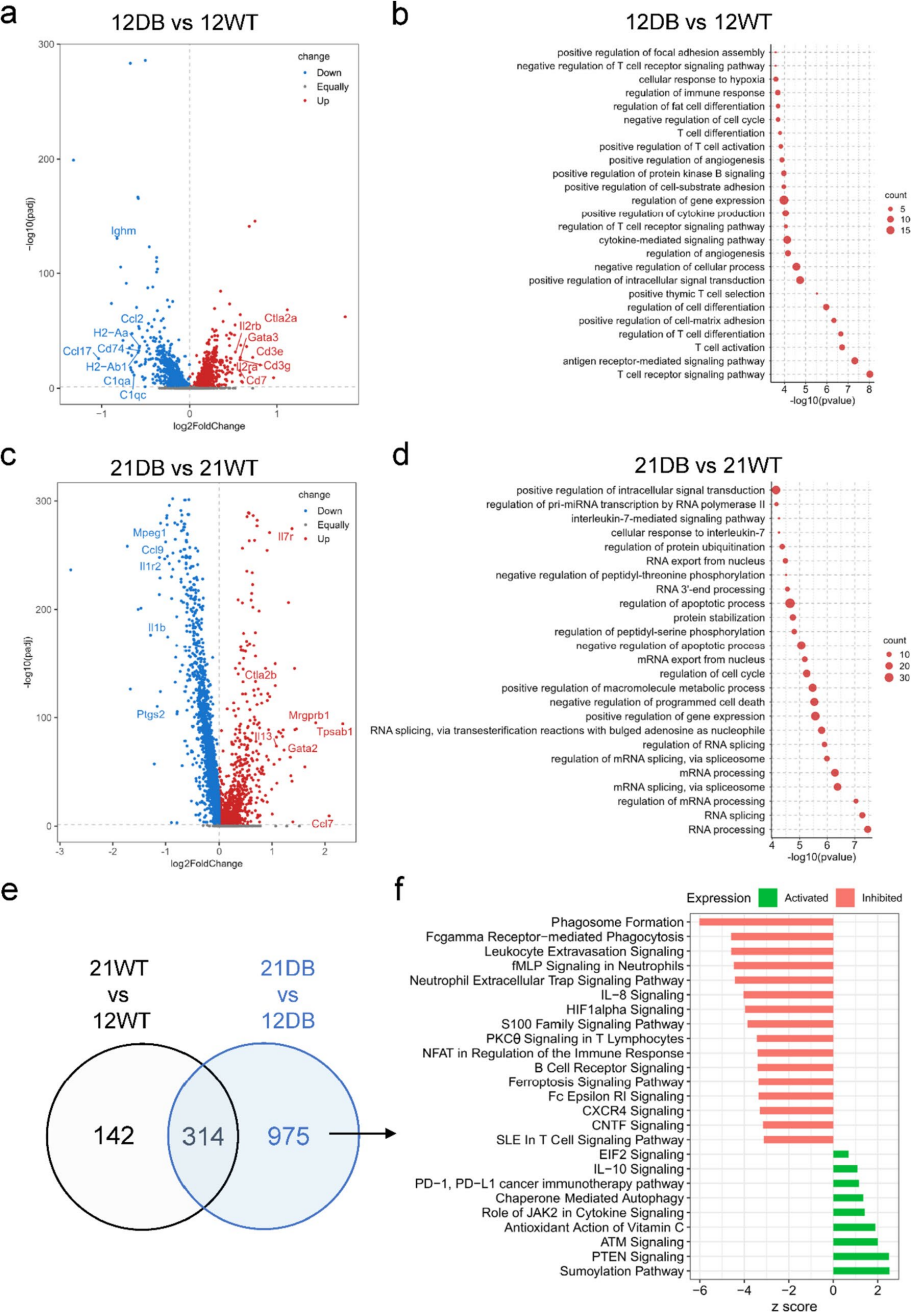
Gene expression and pathway analysis: **a** Volcano plot for the 12-week-DB vs 12-week-WT comparison showing − log10 (*p* value) and log2 fold changes for all transcripts. Those upregulated in 12-week-DB in red, downregulated in 12-week-DB in blue. Gray genes = no statistically significant change in expression. **b** Ingenuity Pathway Analysis (IPA) of upregulated genes from the 12-week-DB compared with 12-week-WT. **c)** Volcano plot for the 21-week-DB vs 21-week-WT comparison showing − log10 (*p* value) and fold changes for all transcripts. Those upregulated in 21-week-DB in red, downregulated in 21-week-DB in blue. Gray genes = no statistically significant change in expression. **d** Pathway analysis for transcripts upregulated in 21-week-DB compared with 21-week-WT. **e** Venn diagram showing the overlapping of the gene expression from the comparison of 12-week-WT vs 21-week-WT and 12-week-DB vs 21-week-DB. **f** Pathway analysis showing the activated or inhibited pathways represented by the 975 differentially expressed genes unique to DB mice

We investigated the functional features of the cells obtained from each group using GO biological process enrichment. We then compared 12-week-WT mice with 12-week-DB mice (Fig. 2a, b) and 21-week-WT mice with 21-week-DB mice (Fig. 2c, d**; **Supplementary Table 4). Comparing 12-week-DB mice with 12-week-WT mice identified T cell-related transcripts (e.g., *Cd3e*, *Cd3g*, *Il2ra*) upregulated in 12-week-DB mice, while transcripts typical of antigen-presenting cells (e.g., *Cd74*, *H2-Aa/Ab1*, *C1qa*) were downregulated. Consistent with these findings, the GO biological process enrichment was dominated by T cell development pathways (T cell differentiation, regulation of T cell receptor signaling, T cell activation), suggesting generalized T cell activation in 12-week-DB mice.

Comparing 21-week-WT with 21-week-DB mice yielded a similar upregulation of T cell-related transcripts (e.g., *Ctla2b*, *Gata3*, *Il13*) and downregulation of proinflammatory innate immune responses (e.g., *Ptgs2*, *Il1b*, *Mpeg1*). However, comparison of 21-week-WT with 21-week-DB mice (Fig. 2c, d) revealed pathways related to RNA processing and gene expression. To further assess chronicity-associated shifts in gene expression in DB mice, we took the 1,289 genes differentially expressed between 12-week-DB and 21-week-DB mice and excluded 314 genes that were also differentially expressed in 12-week-WT versus 21-week-WT mice, resulting in 975 differentially expressed genes specific to DB mice (Fig. 2e, f). Many pathways upregulated in 12-week-DB or 21-week-DB mice spanned multiple cell types. Notably, IL-10, PD-1/PD-L1, and autophagy-related pathways were upregulated in 21-week-DB mice, while phagocytosis, Fc receptor, and B-cell/T cell-related signaling were downregulated.

**Fig. 3:**
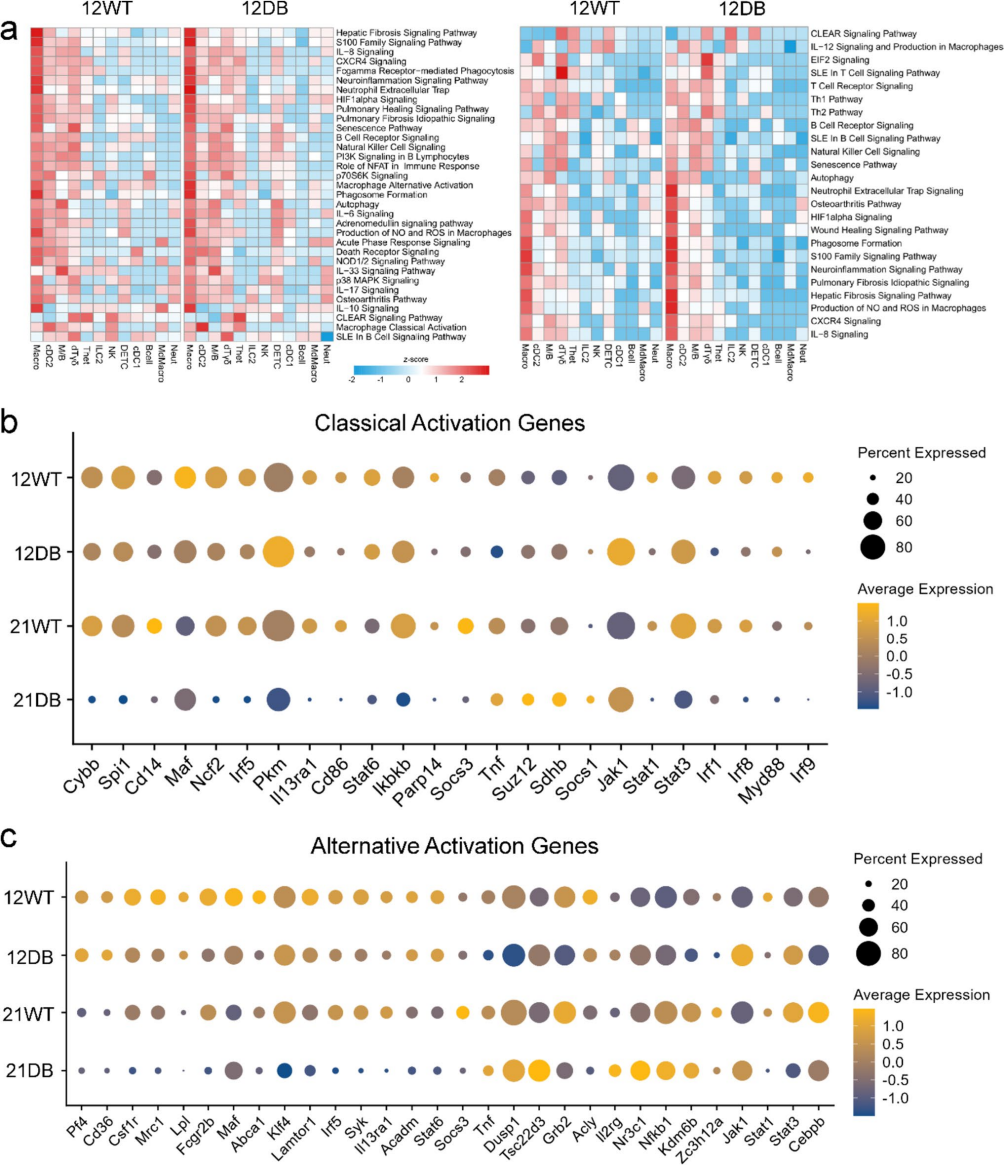
Comparative pathway analysis by cell type. **a** Heatmap showing comparative immune-specific pathway analysis for all cell types from 12-week-WT and 12-week-DB (left) and 21-week-WT and 21-week-DB (right) samples. **b** Dot plot showing the expression of macrophage classical activation and **c** macrophage alternative activation signaling genes

Since our analysis showed a large variation in cell type-specific genes without a clear indication of the cell types responsible, we carried out cluster-specific comparative pathway analysis (Fig. 3). In age-matched WT versus DB comparisons, all samples showed disproportionate upregulation of pathways within the macrophage cluster, with DB mice showing more upregulation compared to WT mice of the same age (Fig. 3a). However, analysis of genes related to classical or alternative macrophage activation was inconclusive, reaffirming the heterogeneity of tissue macrophages (Fig. 3b, c).

### Cell–cell communication shows dysregulation of multiple signaling pathways

**Fig. 4:**
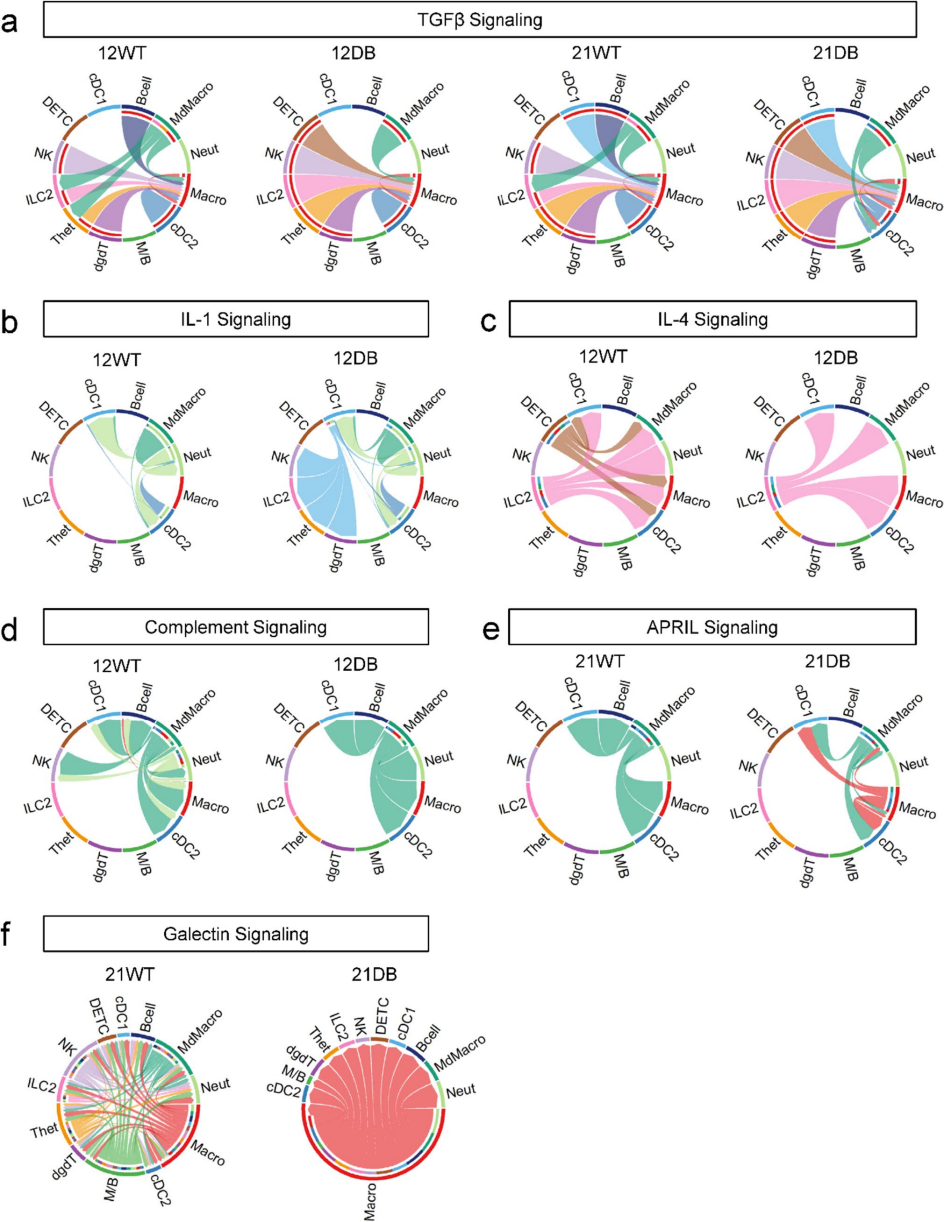
Cell-to-cell communication **a** Chord diagram showing the network of TGFβ signaling in 12-week-WT, 12-week-DB, 21-week-WT, and 21-week-DB. **b** IL-1 signaling in 12-week-WT and 12-week-DB. **c** IL-4 signaling in 12-week-WT and 12-week-DB. **d** Complement signaling in 12-week-WT and 12-week-DB. **e** APRIL signaling in 21-week-WT and 21-week-DB, and **f** galectin signaling in 21-week-WT and 21-week-DB

To generate more insight into the potential dysregulation of immune cell signaling in DB versus WT mice, we undertook an analysis of inferred signaling between the 12 leukocyte clusters across both timepoints (Supplementary Fig. 2). TGFβ is a pleiotropic cytokine with roles in adult tissue homeostasis and fibrosis [36]. In DB mice at 12 and 21 weeks of age, we observed a loss of B-cell-derived TGFβ signaling to macrophages, accompanied by a loss of monocyte-derived macrophage signaling to Type 2 innate lymphoid cells and heterogeneous T cells (Fig. 4a). At 12 weeks of age, we also saw an emergence of signaling related to the proinflammatory cytokine interleukin-1 (IL-1). Signaling from Type 1 classical dendritic cells to T cell-related clusters (natural killer cells, Type 2 innate lymphoid cells, heterogeneous T cells, and delta-gamma T cells) was detected in DB but not WT mice (Fig. 4b). There was also a notable absence of dendritic epidermal T cell-derived interleukin-4 (IL-4) signaling in 12-week-old DB mice, though Type 2 innate lymphoid cell-derived IL-4 signaling was retained (Fig. 4c). Neutrophil-derived complement signaling was also undetectable in 12-week-old DB mice (Fig. 4d). Finally, in 21-week-old DB mice, there was an emergence of macrophage-derived signaling via APRIL (“a proliferation-inducing ligand;” Fig. 4e) and galectin (Fig. 4f) signaling. Collectively, this indicates profound dysregulation of innate and adaptive immune cell signaling networks in DB skin at both ages tested.

### Role of macrophages in T2DM

**Fig. 5:**
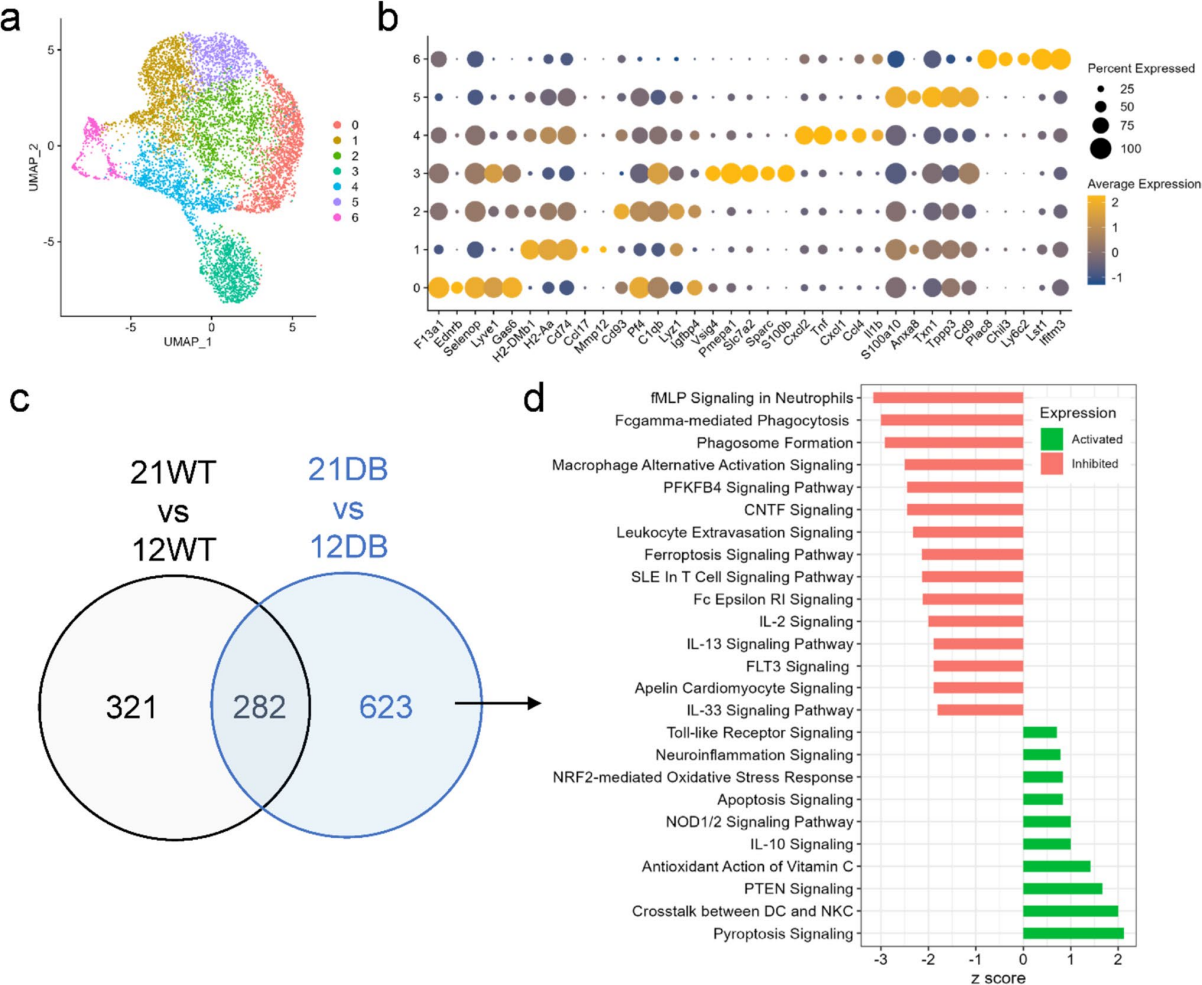
Macrophage sub-clustering analysis. **a** UMAP plot of all 7699 macrophages grouped into 7 sub-clusters. **b** Dot plot showing the top five gene markers from each cluster. **c** Venn diagram showing the overlap of differentially expressed genes from 21-week-WT vs 12-week-WT and 21-week-DB vs 12-week-DB macrophages. **d** The pathway analysis of 623 genes unique to DB showing the activated or inhibited pathways from the 21-week-DB as compared to 12-week-DB

Because of the disproportionate transcriptional upregulation of macrophages (Fig. 3a) and bidirectional changes in macrophage signaling associated with several key cytokines (Fig. 4), we next carried out unsupervised sub-clustering of the macrophage cluster. Unsupervised sub-clustering of macrophages revealed seven distinct clusters based on the expression of markers not consistent with known macrophage populations (Fig. 5a, b and Supplementary Table 5). To determine shifts in macrophage gene expression associated with chronicity of diabetes, we identified the 623 differentially expressed genes that were unique to DB mice (Fig. 5c). Pathway analysis of these genes (Fig. 5d and Supplementary Table 6) showed shifts in similar pathways compared to the comparison made across all cells (Figs. 2f and 5d), including downregulation of N-formyl-methionyl-leucyl-phenylalanine signaling in neutrophils, phagocytosis-related pathways, Fc receptor signaling and upregulation of IL-10, and phosphatase and tensin homolog (PTEN) signaling.

### Lyve1 and MHCII cluster classification

**Fig. 6:**
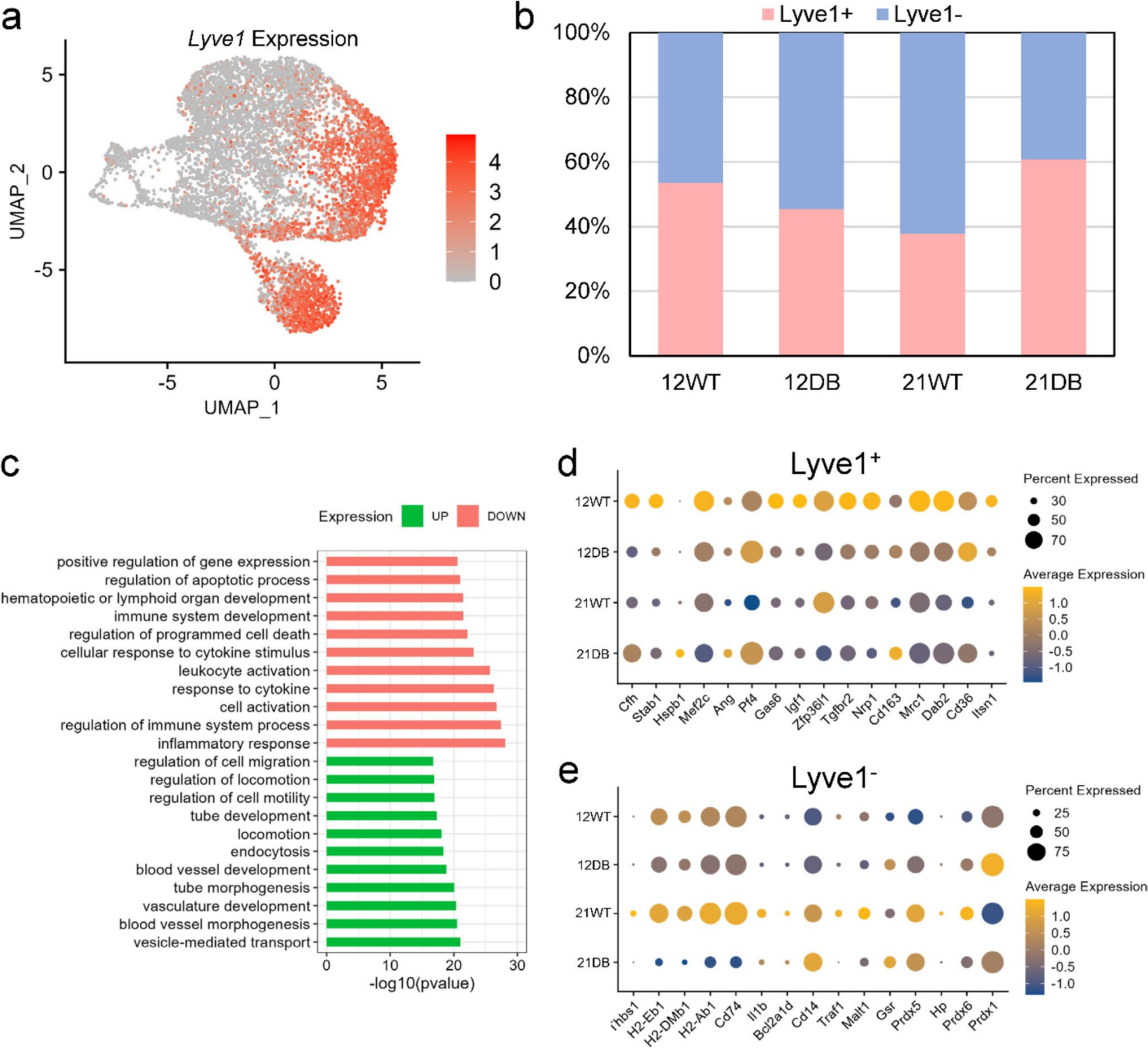
Analysis of Lyve1^+^ and Lyve1^−^ macrophage sub-populations: **a** UMAP plot showing Lyve1 expression across macrophage sub-clusters. **b** Bar plot showing the relative proportion of Lyve1^+^ and Lyve1^−^ cells across samples. **c** GO biological process enrichment analysis of upregulated and downregulated genes from Lyve1^+^ vs Lyve1^−^ macrophages. **d** Dot plot showing the expression pattern of Lyve1^+^ and **e** Lyve1^−^-specific genes across the samples

A new classification system for resident tissue macrophages based on Lyve1 and MHCII expression was recently proposed [37, 38]. Our dataset shows that expression of Lyve1 is clearly delineated, with Lyve1 predominantly expressed in macrophage clusters 0 and 3 (Fig. 6a), and Lyve1^+^ cells representing 40–60% of the total macrophage population, depending on age/genotype (Fig. 6b). Interestingly, 12-week-DB mice had a slightly lower proportion of Lyve1^+^ macrophages than 12-week-WT mice, whereas 21-week-DB mice had approximately 50% more Lyve1^+^ macrophages than 21-week-WT. Pathway analysis of Lyve1^+^ vs Lyve1^−^ macrophages showed that Lyve1 expression was associated with expression of genes related to mobility and vascular development, while Lyve1^−^ macrophages were more dedicated to immune cell function (Fig. 6c). This is consistent with the findings of Krasniewski et al. and Chakarov et al., which suggest Lyve1^+^ macrophages are M2-like and Lyve1^−^ macrophages are M1-like. This is further supported by the gene expression profile (Fig. 6d, e and Supplementary Table 7) which shows that Lyve1^−^ macrophages express transcripts for antigen presentation (*H2-Eb1, H2DMb1, H2-Ab1, CD74*), and Lyve1^+^ macrophages express transcripts related to angiogenesis (*Ang, Stab1, Cfh, Hspb1*), a distinction also noted by Krasniewski et al. [37].

**Fig. 7:**
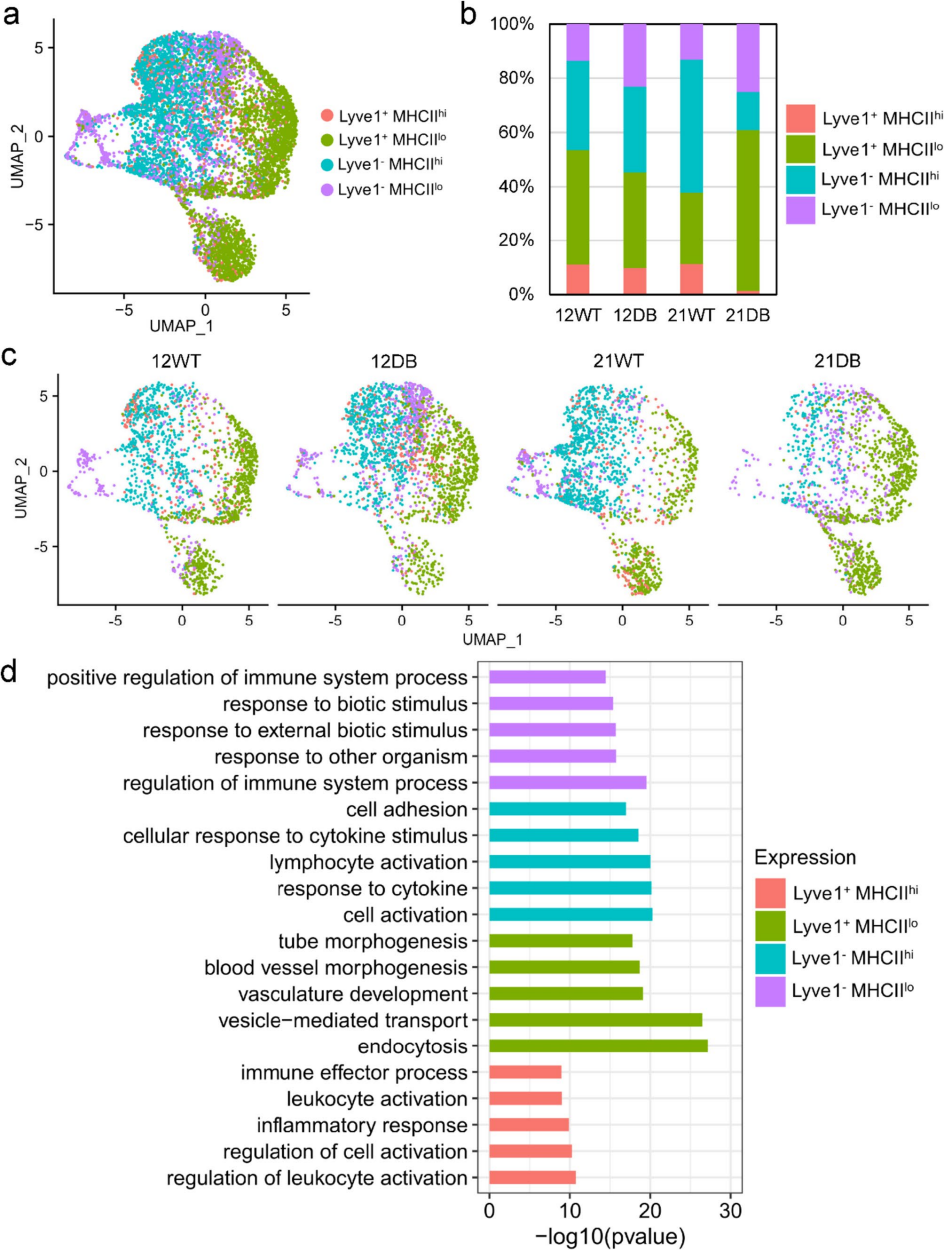
Classification of macrophages into four sub-groups based on Lyve1 and MHCII expression. **a** UMAP projection showing the classification of macrophages based on the expression of Lyve1 and MHCII: Lyve1^+^/MHCII^hi^, Lyve1^+^/MHCII^lo^, Lyve1^−^/MHCII^hi^, and Lyve1^−^/MHCII^lo^. **b** Bar plot showing the relative proportion of the four sub-groups across the samples. **c** UMAP projection of four sub-groups for each sample. **d** GO biological process analysis of differentially expressed genes for each group

Layering MHCII expression onto the expression of Lyve1 permitted formation of four sub-populations [37]: Lyve1^+^ MHCII^hi^, Lyve1^+^ MHCII^lo^, Lyve1^−^ MHCII^hi^, and Lyve1^−^ MHCII^lo^ (Fig. 7a, b). This categorization revealed an increase in the relative proportion of Lyve1^+^ MHCII^lo^ macrophages that was unique to 21-week-DB mice. In addition, the numbers of Lyve1^+^ MHCII^hi^ and Lyve1^−^ MHCII^hi^ cells declined in 21-week-DB mice. This suggests a transition to an anti-inflammatory/angiogenic M2-like phenotype in 21-week-DB mice, whereas WT mice remain relatively stable in this regard as they age (Fig. 7b, c). Pathway enrichment for each sub-population showed distinct expression profiles. Lyve1^+^ MHCII^hi^ macrophages showed elevated expression of immune activation/inflammatory response genes; Lyve1^−^ MHCII^hi^ cells increased the expression of genes related to cytokine responses and lymphocyte activation; Lyve1^−^ MHCII^lo^ cells showed elevated expression of genes related to recognition of foreign organisms, and Lyve1^+^ MHCII^Lo^ cells expressed transcripts related to endocytosis and vascular development (Fig. 7d).

### Histological characterization of DB mouse hindpaws

**Fig. 8:**
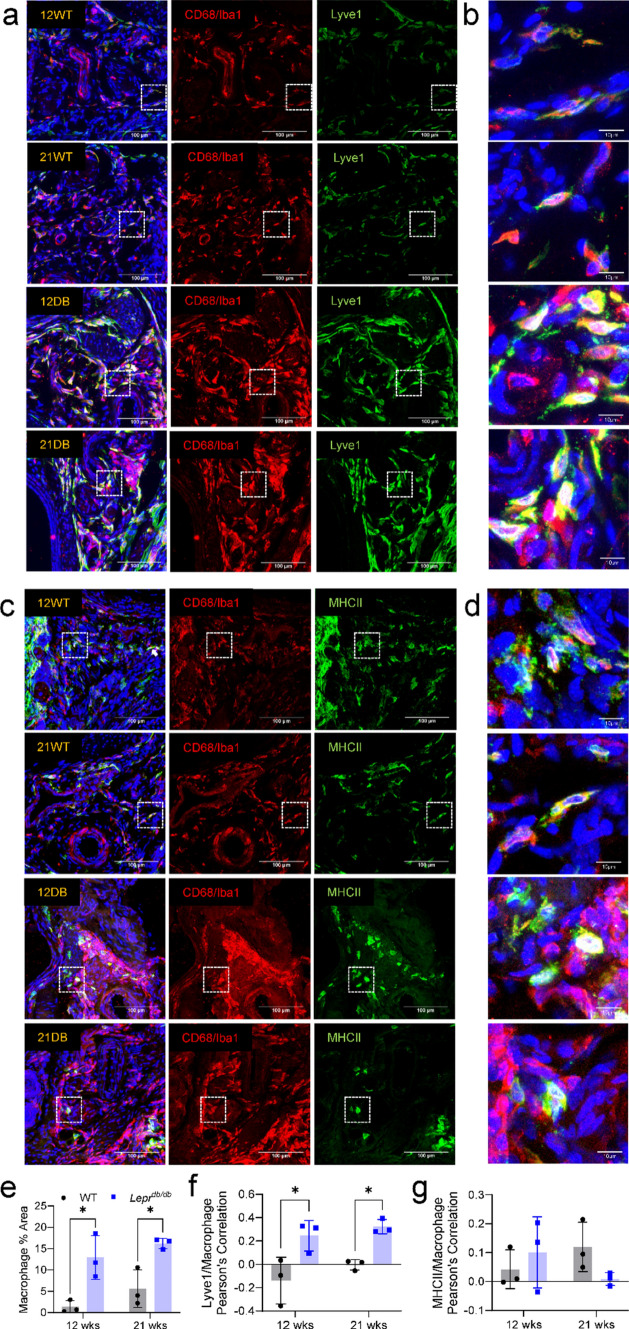
Histological analysis of Lyve1^+^ macrophages in the hindpaws of 12- and 21-week-old aged WT and DB mice. Representative images of CD68/Iba1^+^ macrophages co-stained with **a, b** Lyve1 or **c, d** MHCII antibodies from the hindpaws of 12-week-WT, 21-week-WT, 12-week-DB, and 21-week-DB mice (*n* = 3; dotted white boxes = areas magnified in **b** and** d**. **e** Percent area quantifications for CD68/Iba1^+^ showed a significant increase in macrophage density in DB mice compared to WT groups. **f** This increase in macrophage density corresponded to a significant increase in Pearson’s correlation between CD68/Iba1^+^ signal and Lyve1^+^ signal for 12-week-DB and 21-week-DB mice compared to WT mice. **g** Quantification of MHCII stains showed no significant changes in Pearson’s correlation with CD68/Iba1^+^ signal. *p < 0.05: **p < 0.01. Data are mean ± SD (*n* = 3)

To better understand the distribution of Lyve1^+^ and MHCII^hi^ macrophages within the hindpaw, DB and WT tissues were stained with CD68/Iba1 and co-stained with Lyve1 (Fig. 8a, b) or MHCII (Fig. 8c, d) antibodies. Quantification of CD68/Iba1 immunofluorescence identified a significant increase in macrophage density in the hypodermis of 12-week-DB and 21-week-DB mice compared to their WT counterparts (Fig. 8e). This increase in macrophages was mirrored by a corresponding increase in the correlation between Lyve1 and CD68/Iba1 signal (Fig. 8f), consistent with the emergence of Lyve1^+^ macrophages in DB mice. However, there was no significant change in correlation between MHCII and CD68/Iba1 (Fig. 8g).

**Fig. 9:**
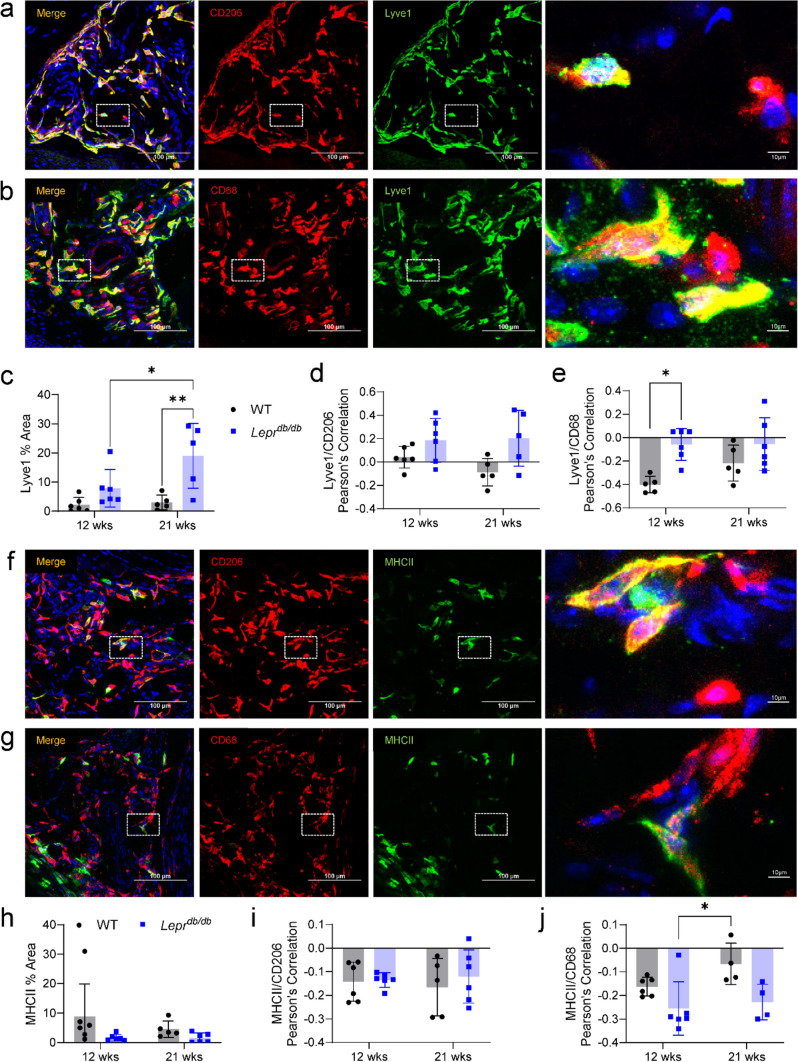
Histological characterization of Lyve1^+^ and MHCII^hi^ macrophages. **a** Representative image of Lyve1 and CD206 colocalization (white dotted box = the area magnified in the right-most image on each row). **b** Representative image of Lyve1 and CD68 colocalization. **c** Quantification of Lyve1^+^ percent area from co-staining with CD206 shows a significant increase in the percent area of Lyve1 in 21-week-DB mice as compared to all other groups. Pearson’s correlation for Lyve1 with **d** CD206 and **e** CD68 revealed a positive correlation between Lyve1 and CD206 and a significant increase in the correlation between Lyve1 and CD68 in the 12- and 21-week-DB mice as compared to 12-week-WT mice. **f** Representative image of MHCII and CD206 colocalization. **g** Representative image of MHCII and CD68 colocalization. **h** Quantification of MHCII percent area from co-staining with CD206 shows no significant differences between groups. Pearson’s correlations for MHCII with both **i** CD206 and **j** CD68 revealed a negative correlation between MHCII and CD206 and between MHCII and CD68, although there was a significantly higher colocalization between MHCII and CD68 within 21-week-WT mice as compared to 12-week-DB mice. *p < 0.05: **p < 0.01. Data are mean ± SD (*n* = 4–6)

From an additional series of colocalization stains, we were able to detect a significant increase in the density of Lyve1^+^ cells in DB mice, which positively correlated with CD206. However, it should also be noted that despite Lyve1 having a somewhat negative correlation with CD68 alone, there was a significant increase in the correlation between CD68 and Lyve1 signals in 12- and 21-week-DB mice as compared to 12-week-WT mice (Fig. 9a–e). In contrast, there was only a moderate non-significant decrease in the percent area for MHCII^+^ cells in 12- and 21-week-DB hindpaws compared to the other groups (Fig. 9f–h), and MHCII had a negative correlation with both CD206 and CD68 (Fig. 9i, j). Alongside quantification and characterization for macrophages, we also determined whether an increase in T cell density was detectable, as the sequencing data suggested (Fig. 2). Immunofluorescent staining of CD3^+^ cells revealed a moderate but non-significant increase in small clusters of T cells within the dermis, suggesting a relatively small area of distribution (Supplemental Fig. 3).

**Fig. 10:**
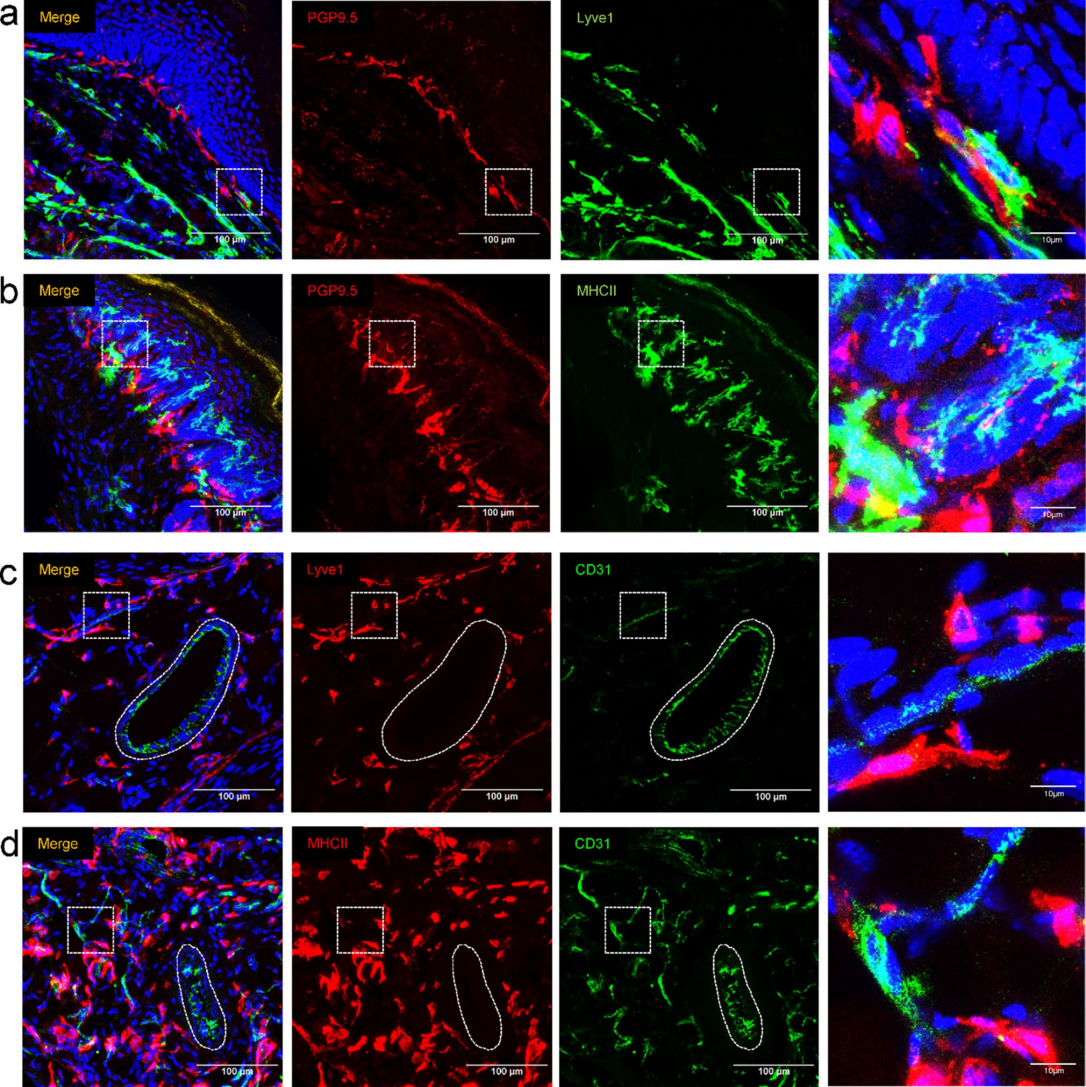
Histological association of Lyve1^+^ and MHCII^hi^ macrophages with nerves and blood vessels. Immunofluorescent staining for **a** Lyve1/PGP9.5 and **b** MHCII/PGP9.5 was used to identify associations of Lyve1^+^ and MHCII^+^ cells with the nerve plexus of the plantar skin (dotted white boxes show the region enlarged in the right column). MHCII^+^ cells showed a much closer association with the nerve plexus as compared to lyve1^+^ cells. A population of MHCII^+^ cells (putative epidermal Langerhans cells, green arrows) was identified in the epidermis. Immunofluorescent staining for **c** Lyve1/CD31 and **d** MHCII/CD31 was used to identify associations of Lyve1^+^ and MHCII^+^ cells with both large vessels (ovoid dotted line) and small vessels (dotted white box) of the hindpaw. Macrophages associated with larger vessels reside near the outer perimeter of the vessel, in the putative tunica externa [39]

In addition to verifying the Lyve1^+^ population as primarily CD206^+^, we also explored the extent to which the Lyve1^+^ or MHCII^+^ population showed close proximity to nerves or blood vessels, since it has been previously reported that both Lyve1^+^ and MHCII^hi^ macrophages are often in close proximity to CD31^+^ blood vessels and TUBB3^+^ nerves [37]. To this end, Lyve1 and MHCII were co-stained alongside PGP9.5 and CD31 to highlight nerves and endothelial cells, respectively. Lyve1^+^ cells reside predominantly in the deeper portions of the dermis, away from the nerve plexus which underlies the epidermis (Fig. 10a), while MHCII^hi^ cells have a strong association with the nerve plexus (Fig. 10b). In addition, we were able to identify Lyve1^+^ and MHCII^+^ cells adjacent to both large and small vessels of the foot (Fig. 10c, d). It should also be noted that we found a distinct population of MHCII^+^ cells in the superficial epidermis, (most likely epidermal Langerhans cells), and that this population also displayed a close association with intraepidermal nerve fibers (Fig. 10a).

## Discussion

The importance of leukocyte dysregulation in the diabetic foot is underlined by the complexity of the role for the immune system in diabetes, DPN, and the formation of foot ulcers [9, 22, 40–44]. However, most transcriptomic analyses in diabetes thus far have focused on the pathophysiology of adipose tissue, sensory ganglia, or wounded/ulcerated skin. Though bulk RNA-sequencing has shown a net increase in proinflammatory gene expression [45], our overall understanding of age- and diabetes-related changes in leukocyte function in the distal limb is incomplete [10, 13, 22, 46]. Here, we analyzed CD45^+^ cells from the hindpaws of 12- and 21-week-old WT and DB mice, which demonstrate both a hyposensitivity and metabolic phenotype [27], to identify disruptions in leukocyte function in the diabetic foot with disease chronicity. Our hope is that a deeper understanding of immune-related disruptions in diabetic hindpaws in the absence of overt lesions will complement studies of proinflammatory cell types associated with bacterial or fungal infections and ulceration.

We identified 12 separate leukocyte clusters with several age- and diabetes-associated shifts in cell populations. The most notable of these included: increased mast cell/basophil, dT_γδ_ cell, heterogeneous T cell, and ILC2 populations in 21-week-DB mice. While these cell types play a host of roles in adaptive and innate immune responses, their presence here may allude to a concerted immune response to the inflamed or hyperglycemic microenvironment. Neutrophils are known to play an early role in the sensory ganglia microenvironment in mouse models of T1DM, and this neutrophilic response is later joined by T cells as the disease progresses [43]. While we did not detect increased neutrophils in diabetic hindpaws, a parallel increase in T cells in diabetes may suggest a larger role for T_het_ cells in late-stage DPN that is generalizable across models and tissues. Consistent with a potential role for T cells in the diabetic foot, our data show that 21-week-DB mice exhibited increased dT_γδ_ cells, and both 12-week-DB and 21-week-DB mice had increased dendritic epidermal T cells (DETCs, which also express the γδ T cell receptor). Interestingly, dT_γδ_ cells and DETCs have been reported to decrease in number and function in diabetic foot ulcers [42, 47]. Since reduced dermal and epidermal T_γδ_ cell numbers impair wound healing, this apparent contradiction might suggest that the mice in our study have not yet reached a point in dermal pathology associated with the development of non-healing wounds. Our data contrast with a study by Taylor et al. in which DB mice exhibited reduced T_γδ_ cell numbers by 10 weeks of age, since we observed increased T_γδ_ cells at 12 weeks (DETC) and 21 weeks (DETC and dT_γδ_ cells) [47]. This apparent discrepancy suggests that further study is needed to determine the underlying factors (e.g., diet, activity levels, immune status) that lead to loss of dermal and epidermal T_γδ_ cells [48]. Interestingly, we found only a moderate increase in the number of CD3^+^ cells via histology, and their distribution appeared in small clusters throughout the dermis. Since T_het_, dermal T_γδ_, and DETC all express CD3, our findings suggest that the changes seen in the single-cell data for these populations are attributable to a comparatively small number of cells. The increase in mast cells/basophils seen in 21-week-DB mice is also consistent with the notion that the mice in our study are not yet in a state conducive to ulceration, since mast cells are known to be important for wound healing [41]. Thus, increased mast cells/basophils (and T cells) may represent a transient inflammatory response in 21-week-DB hindpaws that eventually transitions to loss or senescence of these cells and compromised wound healing. Further examination of these populations at more chronic timepoints (i.e., 12–18 months) is warranted to determine their role in the initiation of DPN and DFU.

The cell–cell communication analysis revealed several changes in cytokine-mediated signaling between leukocytes in DB mice that are likely to be pathologically relevant. Suppressed recruitment of alternatively activated macrophages results in reduced TGFβ levels and impaired wound healing in the streptozotocin-induced model of Type I diabetes and a rat model of Type II diabetes [49, 50]. Similarly, activation of B-cells reduces their production of TGFβ and is associated with a shift away from a more regulatory phenotype [51, 52]. Dendritic cell-derived IL-1 has a stimulatory effect on T cell function in diabetes [53] and the reduced IL-4 signaling from DETCs may induce a net Th1 bias [54]. Impairments in neutrophil complement signaling would be consistent with the increased susceptibility to infection seen in diabetes [55]. Finally, the expression of APRIL by proinflammatory macrophages [56] and the expression of galectin by macrophages within atherosclerotic plaques [57] are consistent with the development of diabetes-related inflammation and cardiovascular disease in this model. Several publications implicate macrophages in the pathogenesis of diabetic neuropathy, with CD68^+^ macrophages detectable in sensory ganglia of patients with T2DM and DPN [10] and inflammatory pathways upregulated in macrophages in adipose tissue, foot ulcers, and circulating monocytes [12, 13, 22, 40, 58]. Here, we identified seven macrophage sub-clusters with a large degree of overlap in the defining genes. However, pathway analysis showed an interesting trend toward activation of Toll-like receptors, oxidative stress, and neuroinflammatory signaling in 21-week-DB mice. Of particular interest is the downregulation of pathways for IL-2 signaling, an important cytokine for proliferation of various T cell sub-types [59]. This may suggest that macrophages in 21-week-DB mice are not primarily responsible for driving the observed increase in T cell/dermal Tγδ cell/DETC/ILC2 numbers.

Based on the inconclusive findings from our initial unsupervised clustering of macrophages, we sought a more appropriate functional classification. A recent report identified a classification system that aligned macrophages with four sub-categories defined by Lyve1 and MHCII [37]. Several recent publications indicate that Lyve1^+^ macrophages represent anti-inflammatory, “M2-like” perivascular macrophages responsible for tissue maintenance. Conversely, MHCII^hi^ is a broad marker for classical/”M1-like” proinflammatory macrophages [58]. However, MHCII is less consistently used as a sole marker since it labels a somewhat heterogeneous subset and can be readily upregulated in response to a wide variety of proinflammatory stimuli [38, 60, 61]. Pathway analysis of these four sub-populations showed that Lyve1^+^MHCII^lo^ expressed genes related to vascular and circulatory system development; Lyve1^−^MHCII^hi^ expressed genes related to lymphocyte activation, cell adhesion, and cytokine-based responses; Lyve1^+^MHC^hi^ upregulated genes related to leukocyte activation and inflammatory responses; and Lyve1^−^MHCII^lo^ expressed genes related to recognition of foreign antigens. This suggests Lyve1^+^MHCII^lo^ macrophages’ responsibility in tissue maintenance and vascular development, while the other three sub-types drive inflammatory responses. In DB mice, it is tempting to speculate that the age-related shift toward the anti-inflammatory, perivascular Lyve1^+^MHCII^lo^ phenotype and shift away from the nerve fiber-associated, Lyve1^−^MHCII^hi^ phenotype drive neuropathy in DB mice [27, 38, 40]. Alternatively, this shift may represent a homeostatic response to the vascular disturbances seen in diabetes.

Histological examination of Lyve1 and MHCII alongside pan-macrophage antibodies suggested a significant increase in the number of macrophages, as well as the presence of Lyve1^+^ macrophages in DB skin. The notion that these Lyve1^+^ macrophages are more “M2-like” appears to be corroborated by the extensive co-localization with another M2-like marker: CD206/Mrc1 [62]. In contrast to the increase seen in Lyve1^+^ macrophages, we noted no significant change in MHCII^+^ macrophages when comparisons were made between each group. Since much of the existing literature considers vascular disturbances as one of the primary mechanisms driving both DPN and DFU [3, 4, 7], a role for angiogenic Lyve1^+^ macrophages in the pathological mechanisms of the diabetic foot could lead to potential markers for disease progression and potent targets for therapeutic development.

Interestingly, while both MHCII^+^ and Lyve1^+^ cells showed a close proximity to small and large blood vessels of the foot, there was a unique spatial separation of the Lyve1^+^ and MHCII^+^ cells relative to the nerve plexus of the skin. More specifically, MHCII^+^ cells showed a close proximity to the nerve plexus and IENFs, whereas Lyve1^+^ cells were predominantly localized to the deep dermis and showed little association with nerves. This contrasts with a previous publication exploring the distribution of Lyve1^+^ and MHCII^+^ cells in skeletal muscle, which showed that these cells were in close proximity to nerves and blood vessels [37].

Through the data shown here, we can conclude that the cutaneous immune system in Type 2 diabetes exhibits profound changes in population and function alongside symptoms of sensory loss, but prior to the development of infection or ulceration. The upregulation of angiogenic, M2-like macrophages under chronic diabetic conditions represents an important functional change in this environment. One of the limitations of this study is that only male mice were used. In future studies, the addition of female *Lepr*^*db/db*^ mice could shed light on sex-based differences in immune dysregulation in diabetes, given the differences in innate immune cell function in males versus females [63]. In addition, although we focused on macrophages due to their disproportionate frequency and transcriptional activity in skin, an in-depth analysis of the various other sub-types leukocytes affected in DB mice was not carried out. In future work, comparison with other models of diabetes, particularly those featuring sensory gain as a model of painful DPN, could prove informative. It will also be important to eventually verify the extent to which these changes are present in diabetic patients, and whether they represent targets for therapeutic intervention to treat DFU and DPN.

### Supplementary Information

Below is the link to the electronic supplementary material.

Supplementary file1 (DOCX 2901 KB)

Supplementary file2 (XLSX 422 KB)

Supplementary file3 (XLSX 774 KB)

Supplementary file4 (XLSX 22 KB)

Supplementary file5 (XLSX 126 KB)

Supplementary file6 (XLSX 192 KB)

Supplementary file7 (XLSX 111 KB)

Supplementary file8 (XLSX 141 KB)

## Data Availability

The datasets generated and analyzed during the current study are available in the Gene Expression Omnibus (GEO) data repository under accession number GSE247211.

## Abbreviations

12DB: 12-Week-old *Lepr*^*db/db*^ mice
12WT: 12-Week-old wild-type mice
21DB: 21-Week-old *Lepr*^*db/db*^ mice
21WT: 21-Week-old wild-type mice
ANOVA: Analysis of variance
AUC: Area under the curve
DAPI: 4',6-Diamidino-2-phenylindole
DB: *Lepr*^*db/db*^ Mouse
cDC1: Type 1 conventional dendritic cells
cDC2: Type 2 conventional dendritic cells
DETC: Dendritic epidermal T cells
DFU: Diabetic foot ulcer
DPN: Diabetic peripheral neuropathy
dTγδ: Dermal gamma delta T cells
FACS: Fluorescence-activated cell sorting
FBS: Fetal bovine serum
FSC: Forward scatter
ILC2: Type 2 innate lymphoid cells
*Lepr*^*db/db*^: Leptin receptor, homozygous for the “db” allele
M/B: Mast cells/basophils
MdMacro: Monocyte-derived macrophages
Neut: Neutrophils
NK: Natural killer cells
PBS: Phosphate-buffered saline
PCA: Principal component analysis
PTEN: Phosphatase and tensin homolog
RT: Room temperature
SSC: Side scatter
T2DM: Type 2 diabetes mellitus
Thet: Heterogeneous T cells
UMAP: Uniform manifold approximation and projection
WT: Wild-type
