# Supplementary material for: Single-cell analysis of age-related changes in leukocytes of diabetic mouse hindpaws
Source: Cell Mol Life Sci. 2024 Mar 19;81(1):146. doi: 10.1007/s00018-024-05128-z (PMC10951029; doi:10.1007/s00018-024-05128-z)
Supplement: Supplementary file 1 — Supplementary file1 (DOCX 2901 KB) [file 18_2024_5128_MOESM1_ESM.docx]

**Single-Cell Analysis of Age-Related Changes in Leukocytes of Diabetic Mouse Hindpaws**

***Cellular and Molecular Life Sciences***

James M. Nichols, DVM PhD^1^, Hoang Vu Pham, MS^1^, Eric Lee^1^, Rajasekaran Mahalingam, PhD^1*^, Andrew J. Shepherd, PhD^1*^

1. The MD Anderson Pain Research Consortium and the Laboratories of Neuroimmunology, Department of Symptom Research, Division of Internal Medicine, The University of Texas MD Anderson Cancer Center, Houston, TX 77030.

*Co-corresponding authors: Rajasekaran Mahalingam, Laboratories of Neuroimmunology, Department of Symptom Research, the University of Texas MD Anderson Cancer Center, Unit 1055, 6565 MD Anderson Boulevard, Houston, Texas 77030. Email: [rmahalingam@mdanderson.org](mailto:rmahalingam@mdanderson.org). Andrew J. Shepherd, Laboratories of Neuroimmunology, Department of Symptom Research, the University of Texas MD Anderson Cancer Center, Unit 1055, 6565 MD Anderson Boulevard, Houston, Texas 77030. Email: [ajshepherd@mdanderson.org](mailto:ajshepherd@mdanderson.org). ORCID ID: 0000-0002-8440-6192.

**
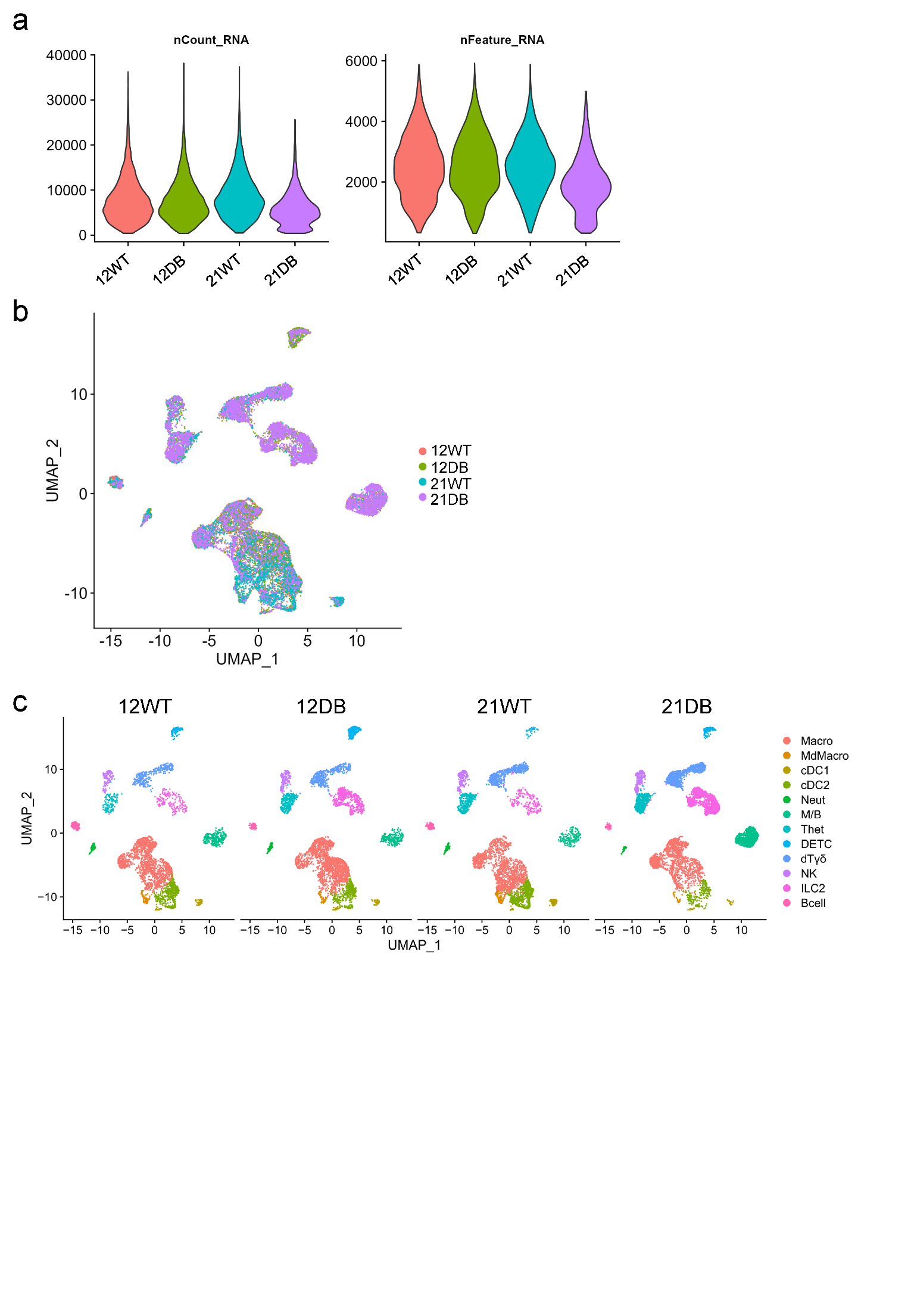
**

**Supplementary Fig. 1** Integrated data UMI counts and UMAP projections. **a)** Violin plot showing the total number of UMI counts and the total number of genes across samples. **b)** Integrated UMAP projection of all four samples. **c)** UMAP plot of each sample with annotated clusters.


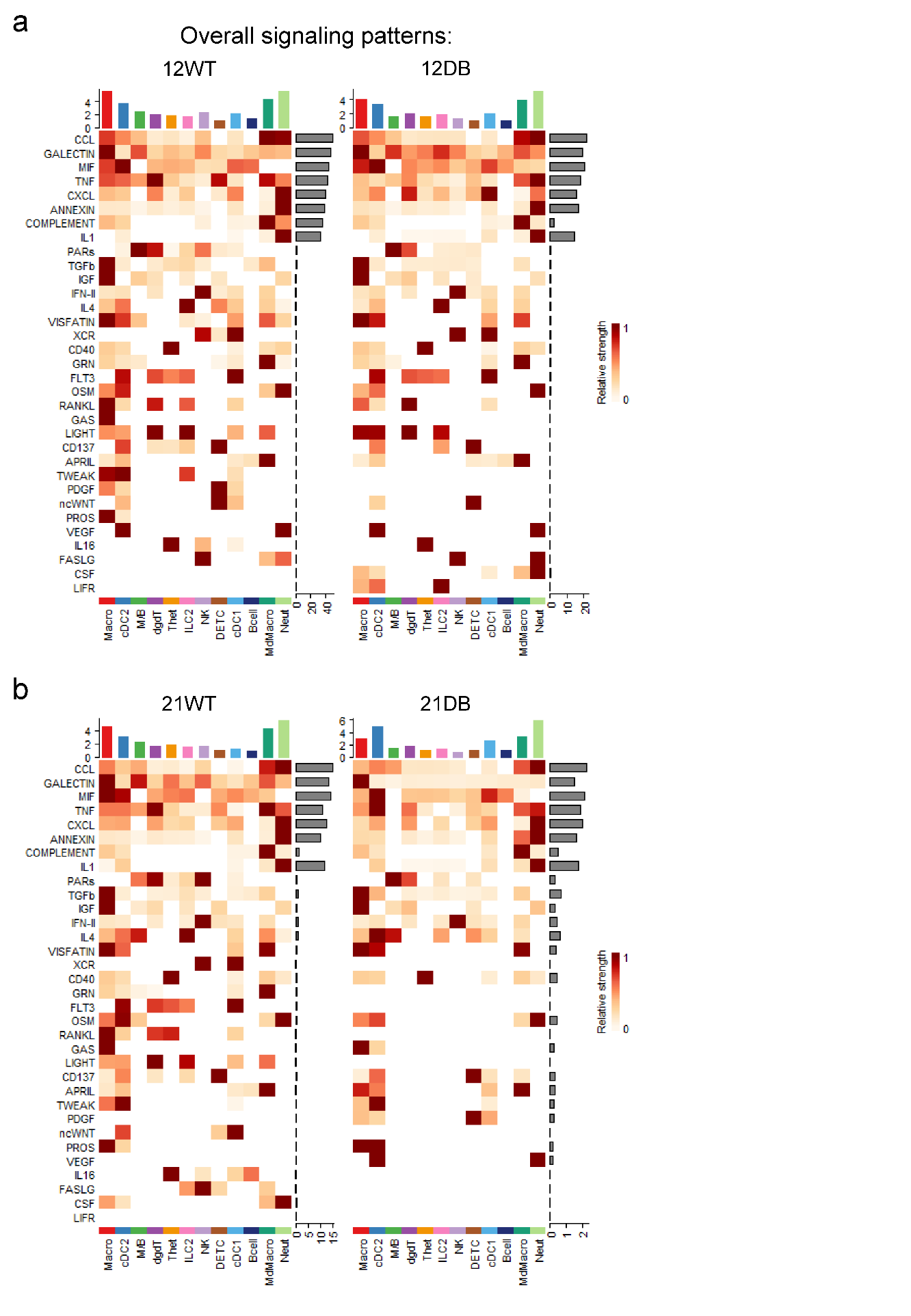


**Supplementary Fig. 2** Heatmaps of overall signaling combining both incoming and outgoing signaling of all cell types from 12WT, 12DB, 21WT and 21DB.


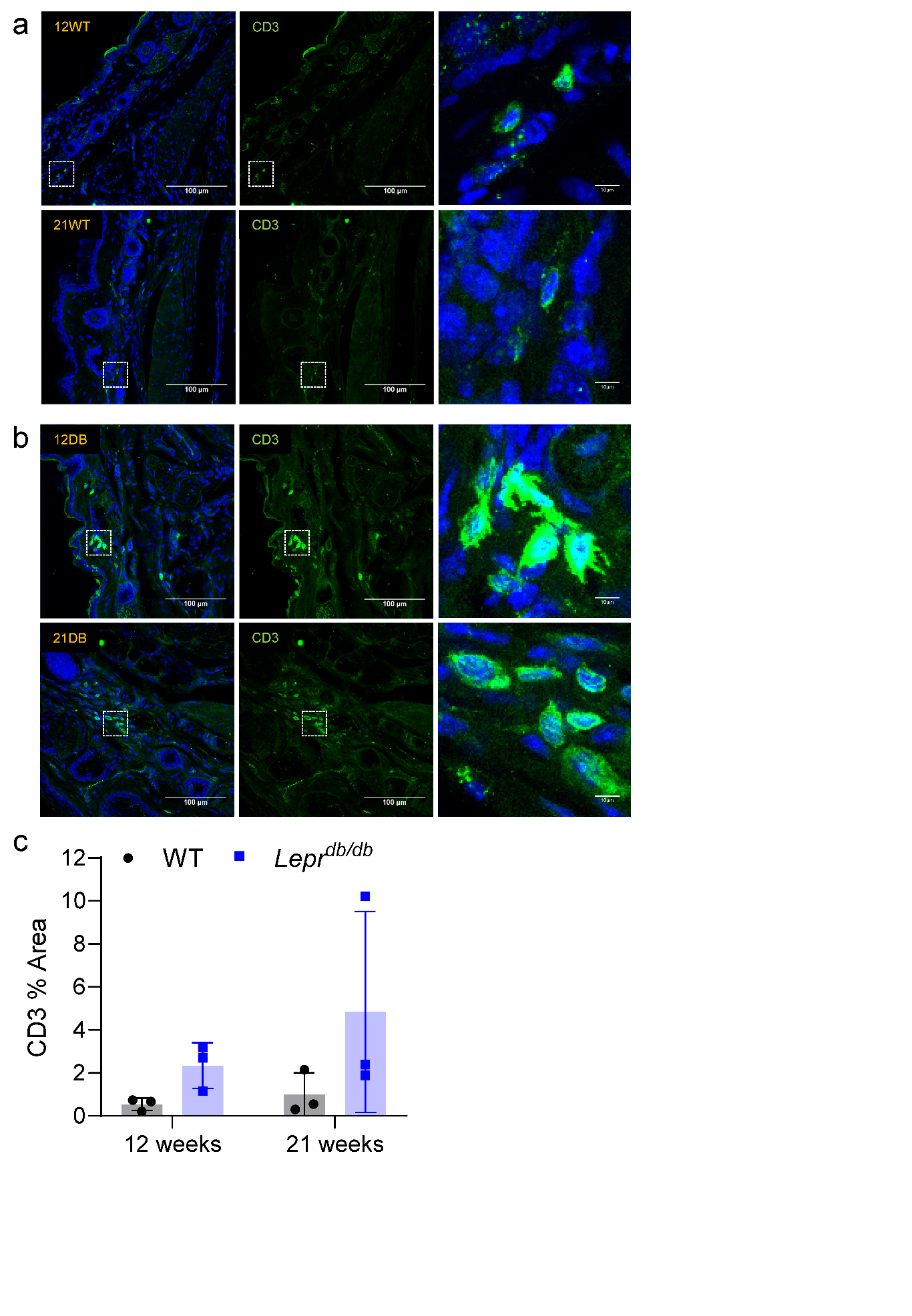


**Supplementary Fig. 3** Histological analysis of CD3^+^ cells in the hindpaws of 12- and 21-week-old WT and DB mice. **a)** Representative images of CD3^+^ cells from the hindpaws of 12-Week-WT, 21-Week-WT, 12-Week-DB, and 21-Week-DB mice (n=3; White dotted box = magnified area shown in right-most panel). **b)** Percent area quantifications for CD3^+^ cell showed no significant difference in T cell density between groups.
